# Phthalate exposure and reproductive hormones and sex-hormone binding globulin before puberty – Phthalate contaminated-foodstuff episode in Taiwan

**DOI:** 10.1371/journal.pone.0175536

**Published:** 2017-04-14

**Authors:** Hui-Ju Wen, Chu-Chih Chen, Ming-Tsang Wu, Mei-Lien Chen, Chien-Wen Sun, Wen-Chiu Wu, I-Wen Huang, Po-Chin Huang, Tzu-Yun Yu, Chao A. Hsiung, Shu-Li Wang, RAPIT group

**Affiliations:** 1 National Institute of Environmental Health Sciences, National Health Research Institutes, Miaoli, Taiwan; 2 Division of Biostatistics and Bioinformatics, Institute of Population Health Sciences, National Health Research Institutes, Miaoli, Taiwan; 3 Department of Public Health, College of Health Sciences, Kaohsiung Medical University, Kaohsiung, Taiwan; 4 Research Center for Environmental Medicine, Kaohsiung Medical University, Kaohsiung, Taiwan; 5 Department of Family Medicine, Kaohsiung Medical University Hospital, Kaohsiung Medical University, Kaohsiung, Taiwan; 6 Institute of Environmental and Occupational Health Sciences, College of Medicine, National Yang Ming University, Taipei, Taiwan; 7 Taipei Hospital, Ministry of Health and Welfare, Taipei, Taiwan; 8 Department of gynecology and obstetrics, Taichung Hospital, Ministry of Health and Welfare, Taichung, Taiwan; 9 Division of Health Policy Translation, Institute of Population Health Sciences, National Health Research Institutes, Miaoli, Taiwan; 10 Department of Public Health, College of Public Health, China Medical University, Taichung, Taiwan; Massachusetts General Hospital, UNITED STATES

## Abstract

**Background:**

In May 2011, a major incident involving phthalates-contaminated foodstuffs occurred in Taiwan. Di-(2-ethylhexyl) phthalate (DEHP) was added to foodstuffs, mainly juice, jelly, tea, sports drink, and dietary supplements. Concerns arose that normal pubertal development, especially reproductive hormone regulation in children, could be disrupted by DEHP exposure.

**Objective:**

To investigate the association between phthalate exposure and reproductive hormone levels among children following potential exposure to phthalate-tainted foodstuffs.

**Methods:**

A total of 239 children aged <12 years old were recruited from 3 hospitals in north, central, and south Taiwan after the episode. Structured questionnaires were used to collect the frequency and quantity of exposures to 5 categories of phthalate-contaminated foodstuffs to assess phthalate exposure in children. Urine samples were collected for the measurement of phthalate metabolites. The estimated daily intake of DEHP exposure at the time of the contamination incident occurred was calculated using both questionnaire data and urinary DEHP metabolite concentrations. Multiple regression analyses were applied to assess associations between phthalate exposure and reproductive hormone levels in children.

**Results:**

After excluding children with missing data regarding exposure levels and hormone concentrations and girls with menstruation, 222 children were included in the statistical analyses. After adjustment for age and birth weight, girls with above median levels of urinary mono-(2-ethyl-5-hydroxyhexyl) phthalate, mono-(2-ethyl-5-oxohexyl) phthalate, and sum of mono-(2-ethylhexyl) phthalate concentrations had higher odds of above median follicle-stimulating hormone concentrations. Girls with above median estimated average daily DEHP exposures following the contamination episode also had higher odds of sex hormone-binding globulin above median levels.

**Conclusions:**

Phthalate exposure was associated with alterations of reproductive hormone levels in girls.

## Introduction

Phthalate esters are widely used in chemical synthesis and are added during the manufacture of many items used in daily life [1, 2], including food packaging, cosmetics, pharmaceuticals, insecticides, polyvinyl chloride (PVC) products, construction materials, and medical products. Phthalate esters are not chemically bound to the polymer and therefore easily evaporate, migrate, or release into air, foodstuffs, and related materials. Exposure to phthalate occurs by ingestion, dermal absorption, inhalation, or contact with medical devices [2].

The hypothalamus–anterior pituitary–gonadal (HPG) axis regulates and controls the balance of reproductive hormone levels in the body. In brief, gonadotropin-releasing hormone (GnRH) neurons in the hypothalamus induce the secretion of GnRH that subsequently stimulates the anterior pituitary to synthesize and release luteinizing hormone (LH) and follicle-stimulating hormone (FSH) to the gonads. The gonads, testes in the male and ovaries in the female, then synthesize and release sex hormones (which are steroids) into the somatic circulation, mainly producing testosterone (TT) in males and estradiol (E2) and progesterone (PG) in females. The HPG axis is controlled under negative feedback mechanisms by systemic sex hormone concentrations that inhibit GnRH secretion in the hypothalamus and by pituitary responsiveness to GnRH. Environmental phthalate diesters are endocrine disruptors that may interfere with the normal function of the HPG axis, resulting in distorted levels of reproductive hormones [3, 4] and potentially producing reproductive and developmental toxicity [5–7].

Phthalate diesters are reported to have antiandrogenic and weak estrogenic effects [8–10]. Among phthalate diesters, di-(2-ethylhexyl) phthalate (DEHP) is the most widely used [11]. Animal studies have shown that DEHP exposure is associated with decreased TT concentration, lowered sperm counts, and abnormal testicular development due to its adverse effect on Leydig cells [12, 13]. In human studies, DEHP has been linked with decreased TT levels in children [14, 15]. Moreover, prenatal DEHP exposure is reportedly associated with a shorter anogenital distance in boys [16] and a lower sperm volume in adolescent males [17] that perhaps is associated with subsequent infertility in adulthood [18]. DEHP may also contribute to a decline in semen quality due to the increased production of semen with abnormal heads [19]. Higher E2 levels and elevated E2/TT ratios were also found in male PVC workers who were exposed to higher urinary DEHP metabolite concentrations [20].

In May 2011, a major phthalates-tainted foodstuffs incident occurred in Taiwan. DEHP and diisononyl phthalate were illegally added to foodstuffs to replace palm oil as the clouding agent [21]. Among foodstuffs, the Taiwan Food and Drug Administration (TFDA) included 5 categories that were contaminated, including tea drinks, fruit beverages, sport drinks, fruit juice or jelly, and dietary supplements in capsule or powder form for use by children [22]. Since this incident occurred, members of the general public have expressed concerns about phthalate exposure and health effects. The Ministry of Health and Welfare (MOHW) (past the Taiwan Department of Health) therefore set up special clinics in 128 hospitals across Taiwan to provide consultations and basic health examinations to people who were wary of possible exposure to phthalate-tainted foodstuffs. Those who might expose to DEHP/DiNP contaminated foodstuffs that announced by TFDA via self-report would be considered with suspected high levels of exposure and were then transferred to 1 of 3 expert hospitals located in the north, middle, and south of Taiwan. Accordingly, the National Health Research Institute (NHRI) of Taiwan also began a project, the Risk Assessment of Phthalate Incident in Taiwan (RAPIT), to investigate the association between DEHP exposure and health effects in persons who were potentially exposed to phthalate-tainted foodstuffs.

Children are in a critical stage of development and are more sensitive than adults to environmental pollutant exposure. Children exposed to endocrine disruptors may have long-term and stronger effects on their health compared to adults. It is crucial for reproductive hormones to be well regulated for normal pubertal development in children. In the present study, we investigated the association between phthalate exposure and reproductive hormone levels in children who participated in the RAPIT study.

## Materials and methods

### Participant recruitment

**Fig 1:**
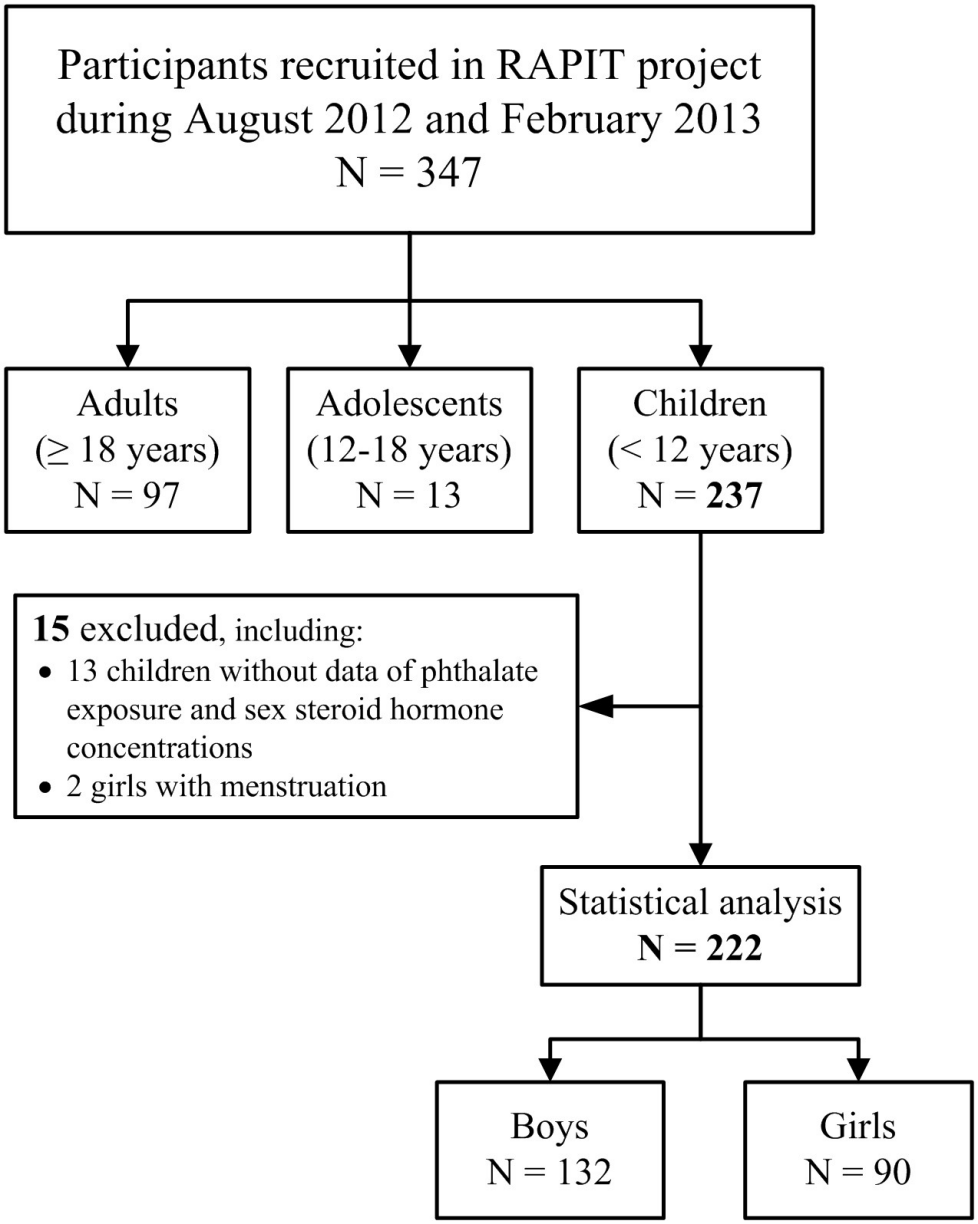
Flow chart of participant recruitment.

The study subjects were recruited from the RAPIT Project 2011 at 3 expert hospitals, the Ministry of Health and Welfare Hospital in Taipei and Taichung and the Kaohsiung Medical University Hospitals. A total of 347 participants, including 237 children (<12 years old), 13 adolescents (12–18 years old), and 97 adults (≥18 years old) were invited to participate during the period from August 2012 to February 2013 (Fig 1). Subjects or their parents gave informed consent and filled out an exposure assessment questionnaire about phthalate exposure; urine and blood samples were collected for phthalate metabolite and biochemical marker measurement; and participants received physical examinations. Children who provided written consent from both their main caretaker and themselves were our major subjects and were recruited into the present study. The study process was approved by the Research Ethnics Committee of the National Health Research Institutes.

### Questionnaire

A structured questionnaire was used to collect information about the frequency, duration, and types of daily exposure to 5 major phthalate-contaminated foodstuffs as announced by TFDA. Questionnaires were also used to collect demographic data, diet, histories of atopic disease (asthma, allergic rhinitis, or atopic dermatitis), health status, and indoor environmental conditions.

### DEHP metabolites and reproductive hormone measurement

Urine samples of participants were collected and stored in brown glass bottles (National Scientific Supply Company, Claremont CA, USA). We conducted blank tests to ensure that the bottles did not contribute to measure phthalate contaminants. The metabolites of DEHP in spot urine, including mono-(2-ethylhexyl) phthalate (MEHP), mono-(2-ethyl-5-hydroxyhexyl) phthalate (MEHHP), and mono-(2-ethyl-5-oxohexyl) phthalate (MEOHP), were measured by high-performance liquid chromatography–tandem mass spectrometry (HPLC-MS/MS) as described in a previous study [23, 24, 25]. The water (Sigma-Aldrich, Switzerland) as blank sample was measured simultaneously in every experiment. The measured concentration of blank sample below twofold of the detection limit value for each phthalate metabolite are as standard of quality control. The experiment was performed in the laboratory at National Institute of Environmental Health Sciences, National Health Research Institutes, Taiwan. For external quality assurance of phthalate metabolite measurement, we annually participated in the intercomparison programme in the German External Quality Assessment Scheme for Biological Monitoring (G-EQUAS). Urinary creatinine levels were measured at Union Clinical Laboratory (UCL; Taipei, Taiwan) using an ADVIA 1800 Clinical Chemistry System (Siemens, Erlangen, Germany). Phthalate metabolite measurements were divided by urinary creatinine levels and expressed as “μg/g creatinine” to account for urinary volume correction.

Reproductive hormones in venous blood were also assessed by UCL. LH (mIU/mL), FSH (mIU/mL), E2 (pg/mL), and TT (ng/dL) levels were measured using a chemiluminescence immunoassay (Centaur; Siemens). Free TT (ng/dL) and sex hormone–binding globulin (SHBG, nmol/L) were assessed using an electrochemiluminescence immunoassay (ECLIA) (Elecsys 2010; Roche, Basel, Switzerland). The detection limit values of MEHP, MEHHP, MEOHP, LH, FSH, E2, TT, free TT, and SHBG were 0.7 ng/mL, 0.1 ng/mL, 0.1 ng/mL, 0.07 ng/dL, 0.3 IU/mL, 11.8 pg/mL, 10.0 IU/mL, 0.04 ng/dL, and 0.35 nmole/L, respectively. The participants’ concentrations of DEHP metabolites and reproductive hormones below the detection limit value were replaced by half-of-detection-limit values.

### DEHP exposure assessment

Participants’ levels of DEHP exposure were estimated by their urinary concentrations of 3 DEHP metabolites and an exposure assessment questionnaire, as described in a previous study [25]. Briefly, the concentration of urinary DEHP metabolites was considered to be the current background DEHP exposure status and was used to calculate the daily intake of DEHP (AvDI_*env*_) (Eq 1).

$$AvDIenv\;\;\left(\mu g/kg\;bw/day\right)\;\;=\;\frac{UE_{sum}\;\;\left(\mu mol/g\right)\times CE\left(g/day\right)}{F_{UE}\times BW\left(kg\right)}\times MW_{DEHP}$$ (1)

UE_*sum*_: Sum of three urinary creatinine-adjusted DEHP metabolite concentrations;

CE: Sex-specific body height-based reference values for urinary creatinine excretion in children < 12 years old;

F_*UE*_: Molar fraction of excreted metabolite relative to total intake at 24-h post-dosing;

BW: Body weight;

MW_*DEHP*_: Molecular weight of DEHP.

Participants’ DEHP exposure to contaminated food products was estimated and reconstructed from the exposure assessment questionnaire, combined with estimated DEHP levels in 2449 foodstuffs reported by the TFDA and the Bureau of Health of Kaohsiung City. We arrived at these estimates by Bayesian models using Markov chain Monte Carlo simulation [25]. Based on the structured questionnaire for 5 major phthalate-contaminated foodstuffs and the self-reported exposure history with more specific detailed information about foodstuffs consumed, we estimated and calculated the AvDI of DEHP exposure levels through contaminated foodstuffs from the questionnaire (AvDI_*QN*_) and the self-report (AvDI_*SF*_) using Eq 2, respectively. Finally, the total daily DEHP exposure (AvDI_*all*_) of participants was estimated as the sum of these 3 estimated levels obtained from urine samples and the questionnaire as Eq 3. If exposure to contaminated foodstuffs from self-report was available in that category, the AvDI_*QN*_ of such food category was counted as zero to avoid double counting and to lower uncertainty [25].

$$AvDI\;\;\left(\mu g/kg\;bw/day\right)\;=\frac{\sum_{i=1}^{5}Y_{i}\times M_{i}\times EF_{i}\times ED}{AT\times BW_{b}}$$ (2)

*i* = 1,…,5; five categories of major phthalate-contaminated foodstuffs;

Y_*i*_ (mg/L or ppm): DEHP concentration;

M_*i*_ (mL or g): amount consumed;

EF_*i*_ (times*day-1): exposure frequency;

ED_*i*_ (day): exposure duration

AT (day): average time of exposure

BW_*b*_ (kg): body weight on May 31, 2011 (the official last day of the incident according to MOHW).

$$AvDI_{all}\left(\mu g/kg\;bw/day\right)=\;AvDI_{ENv}+AvDI_{QN}+AvDI_{SF}$$ (3)

Because there was a time lag of more than one year between the phthalate incident and participant’s recruitment, an additional estimated exposure level, AvDI_*all_wp*_ (μg/kg bw/day), was used to account for the window period between May 31, 2011, and the date of participant recruitment; this value was calculated by replacing AT (day) with AT_*wp*_ (day), which included an extra time lag for urinary DEHP metabolite measurement, and we also replaced the body weight value(BW_*b*_) (kg) with the body weight at recruitment (BW_*p*_) (kg) [25].

### Statistical analysis

Data analysis was done with JMP version 10.0 (SAS Institute, Cary, NC, USA) and SPSS version 20.0 (IBM, Armonk, NY, USA). Because boys and girls may have different levels of reproductive hormone concentrations, children were stratified by sex for statistical analysis. Analysis of variance (ANOVA), nonparametric tests, and *χ*^2^ tests were applied as appropriate to different groups. Spearman’s rank correlation coefficient was applied to assess the correlation between DEHP exposure parameters and reproductive hormone concentrations in children. Multiple logistic regression was also applied to test the association between DEHP exposure parameters and reproductive hormone concentrations in children. All concentrations of DEHP exposure parameters and reproductive hormones were log-transformed to approximate normality. Because multiple comparisons were done to examine the relationships between DEHP exposure and reproductive hormones, the α value was adjusted, and a *p* value ≤ 0.0071 (ie, 0.05 divided by 7) was considered statistically significant.

## Results

After exclusion of 2 girls with menstruation and 15 children without AvDI or reproductive hormone data, a total of 222 children including 132 boys and 90 girls < 12 years old were recruited into the present study (Fig 1). The characteristics of participants and sex differences are shown in Table 1. No significant differences were found between boys and girls in the cohort in terms of body mass index, age, parental education levels, and birth order. Among boys, the development of pubic hair and external genitalia were all at Tanner Stage I. Among girls, 1.1% were at pubic hair Tanner Stage II. For breast development, 12.2% and 1.1% of girls were in Tanner Stages II and III, respectively (Table 1).

**Table 1:** Statistical description of participants’ characteristics by sex.

Variables	All (n = 222)	Boys (n = 132)	Girls (n = 90)	*p* value^¶^
	Mean (SD) or n (%)			
Age (years)^§^	5.48 (2.28)	5.30 (2.22)	5.75 (2.33)	0.144
BMI (kg/m^2^)^§^	16.22 (2.27)	16.26 (2.13)	16.17 (2.47)	0.769
Gestation (weeks)^§^	38.33 (2.16)	38.23 (2.34)	38.49 (1.86)	0.384
Birth weight (kg)^§^	3123.6 (545.3)	3142.2 (539.3)	3096.4 (555.7)	0.541
Birth order				
1st	146 (65.5)	88 (66.2)	58 (64.4)	0.525
≥ 2nd	63 (28.4)	35 (26.5)	28 (31.1)	
ETS exposure during pregnancy				
Yes	44 (19.7)	27 (20.3)	17 (18.9)	0.753
No	177 (79.7)	104 (78.8)	73 (91.1)	
Mother was active smoker during pregnancy				
Yes	5 (2.2)	3 (2.3)	2 (2.2)	0.973
No	216 (97.3)	128 (97.0)	88 (97.8)	
Maternal education				
≤ 12 years	0	0	0	
> 12 years	209 (94.1)	123 (93.2)	86 (95.6)	—^&^
Paternal education				
≤ 12 years	1 (0.4)	1 (0.7)	0	—^&^
> 12 years	208 (93.7)	122 (92.4)	86 (95.6)	
Tanner stage				
Pubic hair				
Stage I		132 (100.0)	89 (98.9)	—^&^
Stage II		0	1 (1.1)	
Breast development				
Stage I		—^&^	78 (86.7)	—^&^
Stage II		—^&^	11 (12.2)	
Stage III		—^&^	1 (1.1)	
Development of external genitalia				
Stage I		132 (100.0)	—^&^	—^&^

^¶^p value was calculated by χ^2^ test for categorical variables and Kruskal–Wallis tests for continues variables as compared between boys and girls.

^§^Mean (SD).

^&^”—”indicates no analysis.

ETS: environmental tobacco smoke.

Some numbers do not add up to total n because of missing values.

Urinary phthalate metabolite concentration was corrected by urinary creatinine levels. Children without urinary creatinine data due to insufficient urine sample would be therefore excluded in analyses of urinary metabolites. There was no significant different children included and excluded in sex, gestational week, and birthweight. However, the excluded children were younger than those included (mean = 2.81 years v.s. 5.68 years, respectively). The distribution of urinary DEHP metabolite concentrations, estimated DEHP exposure levels (AvDI), and reproductive hormone concentrations are reported in Table 2. The concentrations of urinary MEHP and ΣMEHP were significantly higher in boys than in girls. Boys also had higher levels of background DEHP exposure (AvDI_*env*_) than girls. However, higher FSH, E2, and free TT levels were measured in girls than boys (Table 2). The percentages of children with above the detection limit values of phthalate metabolites and reproductive hormones were also reported in Table 2.

**Table 2:** Concentration of urinary DEHP metabolites, estimated DEHP exposure, and reproductive hormones in participants by sex (n = 222).

		Boys (n = 132)						Girls (n = 90)					p-value^¶^
	n	GM (GSD)	AM (SD)	Median	IQR	% > DL	n	GM (GSD)	AM (SD)	Median	IQR	% > DL	
**Urinary DEHP monoesters (μg/g creatinine)**													
MEHP	123	9.05 (0.48)	14.59 (15.14)	10.56	5.35–18.83	100	86	5.82 (0.6)	11.11 (10.95)	7.93	2.66–15.57	100	0.035
MEHHP	122	47.94 (0.33)	64.18 (62.74)	51.51	30.80–68.85	100	86	42.89 (0.27)	51.57 (36.25)	38.88	30.64–61.76	100	0.095
MEOHP	121	32.88 (0.42)	44.83 (38.74)	34.41	21.77–49.91	100	85	28.52 (0.39)	36.58 (27.71)	28.59	22.08–40.24	100	0.089
∑MEHP	121	94.96 (0.32)	124.0 (111.9)	101.7	61.77–135.9	100	85	81.97 (0.27)	98.5 (69.64)	78.53	58.18–105.2	100	0.026
**Estimated DEHP exposure (μg/kg bw/day)**													
AvDIenv	132	6.86 (0.32)	8.99 (8.30)	7.2	4.55–10.2	—^&^	90	6.0 (0.26)	7.09 (4.56)	5.8	4.28–8.30	—^&^	0.039
AvDIall	132	30.0 (0.41)	47.92 (58.79)	29.65	16.58–51.95	—^&^	90	26.96 (0.42)	44.01 (53.6)	25.85	13.78–47.78	—^&^	0.41
AvDIall_wp	132	16.6 (0.37)	25.23 (34.53)	16.25	10.0–25.2	—^&^	90	15.99 (0.35)	22.88 (24.89)	13.2	9.28–27.83	—^&^	0.651
**Reproductive hormones**													
LH (mIU/mL)	132	0.053 (0.37)	0.10 (0.18)	0.035	0.035–0.035	23.5	90	0.051 (0.48)	0.25 (1.09)	0.035	0.035–0.035	14.4	0.142
FSH(mIU/mL)	132	0.91 (0.3)	1.13 (0.75)	0.9	0.6–1.40	95.5	90	2.08 (0.23)	2.38 (1.33)	2.0	1.6–2.83	100	<0.0001
E2 (pg/mL)	132	8.05 (0.24)	9.63 (7.02)	5.9	5.9–12.75	26.5	90	9.54 (0.26)	11.69 (9.94)	5.9	5.9–14.93	44.4	0.021
TT (ng/dL)	132	5.54 (0.15)	6.02 (3.63)	5	5.0–5.0	9.1	90	6.18 (0.22)	7.42 (6.46)	5.0	5.0–5.0	16.7	0.081
Free TT (ng/dL)	132	0.069 (0.19)	0.08 (0.058)	0.06	0.05–0.08	100	90	0.087 (0.24)	0.11 (0.09)	0.07	0.06–0.11	100	0.0003
SHBG (nmol/L)	132	121.3 (0.19)	131.2 (46.79)	137.2	95.35–165.8	100	90	104.8 (0.19)	113.8 (43.01)	109.6	80.9–145.4	100	0.006

^¶^p-value was calculated by Kruskal-Wallis tests.

Some numbers do not add up to total n because of missing values.

^&^"—" was indicated not applicable.

Abbreviations: DEHP, di-(2-ethylhexyl) phthalate; MEHP, mono-(2-ethylhexyl) phthalate; MEHHP, mono-(2-ethyl-5-hydroxyhexyl) phthalate; MEOHP, mono-(2-ethyl-5-oxohexyl) phthalate; AvDIall, estimated daily intake of DEHP exposure; AvDIall_wp, AvDIall with window period; LH, luteinizing hormone; FSH, follicle-stimulating hormone; E2, estradiol; TT, testosterone; SHBG, sex hormone-binding globulin; GM (GSE), geometric mean (geometrical standard error); AM (SE), arithmetic mean; IQR, interquartile range; DL, detection limit value.

We suggested that birthweight was reflected by the growth and development of the newborn which may be associated with later health status. We analyzed the association between covariables and reproductive hormones. Birthweight tends to correlated with reproductive hormone with borderline significant for TT in girls (n = 90) as shown in S1 Table. We therefore adjusted for birthweight in statistical analyses. Table 3 shows Spearman’s correlation between DEHP exposure and reproductive hormone concentrations stratified by sex. Among children, no significant association was found between urinary DEHP metabolite concentrations and reproductive hormone levels after adjustment for age and birthweight. In terms of estimated DEHP exposure levels in children, when we considered the window period between the exposure incident and participants’ recruitment to our study, AvDI_*all_wp*_ was positively correlated with SHBG concentrations in girls (β = 0.276, *p* = 0.009) after adjustment for birth weight and age (Table 3). The result of a sensitivity analysis excluding 4 children suspected to have particular unreliable estimates for AvDI at the episode was shown in S2 Table. The results were similar with or without the exclusions.

**Table 3:** Spearman's correlation between urinary DEHP monoester and estimated DEHP exposure levels and reproductive hormone concentrations in children by sex (n = 222).

	LH (mIU/mL)		FSH (mIU/mL)		E2 (pg/mL)		TT (ng/dL)		FreeTT (ng/dL)		SHBG (nmol/L)	
	Un-adj	adj	Un-adj	adj	Un-adj	adj	Un-adj	adj	Un-adj	adj	Un-adj	adj
Boys												
Urinary DEHP monoesters (μg/g creatinine) (n = 121)												
MEHP	-0.048	-0.040	-0.015	0.022	-0.108	-0.089	-0.038	-0.034	0.069	0.104	-0.134	-0.180*
MEHHP	-0.181*	-0.112	-0.066	0.059	-0.035	-0.018	-0.138	-0.044	-0.082	0.035	0.040	-0.103
MEOHP	-0.172^#^	-0.080	0.002	0.101	-0.118	-0.024	-0.097	-0.006	-0.053	0.042	0.021	-0.087
ΣMEHP	-0.183*	-0.103	-0.042	0.088	-0.087	-0.049	-0.127	-0.024	-0.028	0.060	-0.014	-0.125
Estimated DEHP exposure levels (μg/kg bw/day) (n = 131)												
AvDI_*env*_	-0.077	-0.083	0.041	0.095	-0.027	-0.017	-0.022	-0.033	0.018	-0.015	-0.038	-0.023
AvDI_*all*_	-0.086	-0.056	-0.152^#^	-0.098	-0.073	-0.059	-0.165^#^	-0.008	-0.228**	-0.100	0.242**^§^	0.098
AvDI_*all_wp*_	-0.069	-0.074	-0.065	0.015	-0.080	-0.053	-0.119	-0.025	-0.104	-0.067	0.114	0.057
Girls												
Urinary DEHP monoesters (μg/g creatinine) (n = 86)												
MEHP	0.155	0.073	0.242*	0.186^#^	0.048	-0.025	0.007	-0.013	0.092	0.139	0.063	0.066
MEHHP	-0.138	-0.005	0.120	0.097	0.053	0.121	-0.277**	-0.027	-0.151	0.122	0.241*	0.216^#^
MEOHP	-0.136	-0.149	0.191^#^	0.014	-0.007	-0.013	-0.262*	-0.110	-0.122	0.022	0.214*	0.147
ΣMEHP	-0.114	-0.024	0.164	0.122	-0.002	0.077	-0.256*	-0.027	-0.121	0.140	0.214*	0.187^#^
Estimated DEHP exposure levels (μg/kg bw/day) (n = 90)												
AvDI_*env*_	-0.075	-0.026	0.119	0.067	0.026	0.051	-0.239*	-0.073	-0.169	0.053	0.275**	0.266*
AvDI_*all*_	-0.162	0.029	-0.061	0.013	-0.073	-0.021	-0.177^#^	-0.054	-0.251*	-0.079	0.334**^§^	0.233*
AvDI_*all_wp*_	-0.141	0.006	-0.016	0.017	-0.085	-0.031	-0.177^#^	-0.045	-0.288**^§^	-0.102	0.384***^§^	0.276**

^¶^Adjusted for age and birth weight.

^#^p<0.1,

*p<0.05,

**p<0.01,

***p<0.001,

^§^p<0.0071 indicates a statistical significant correlation.

All concentrations of urinary DEHP monoester, estimated DEHP exposure, and reproductive hormones were log 10 transformed.

Some numbers do not add up to total n because of missing values.

**Fig 2:**
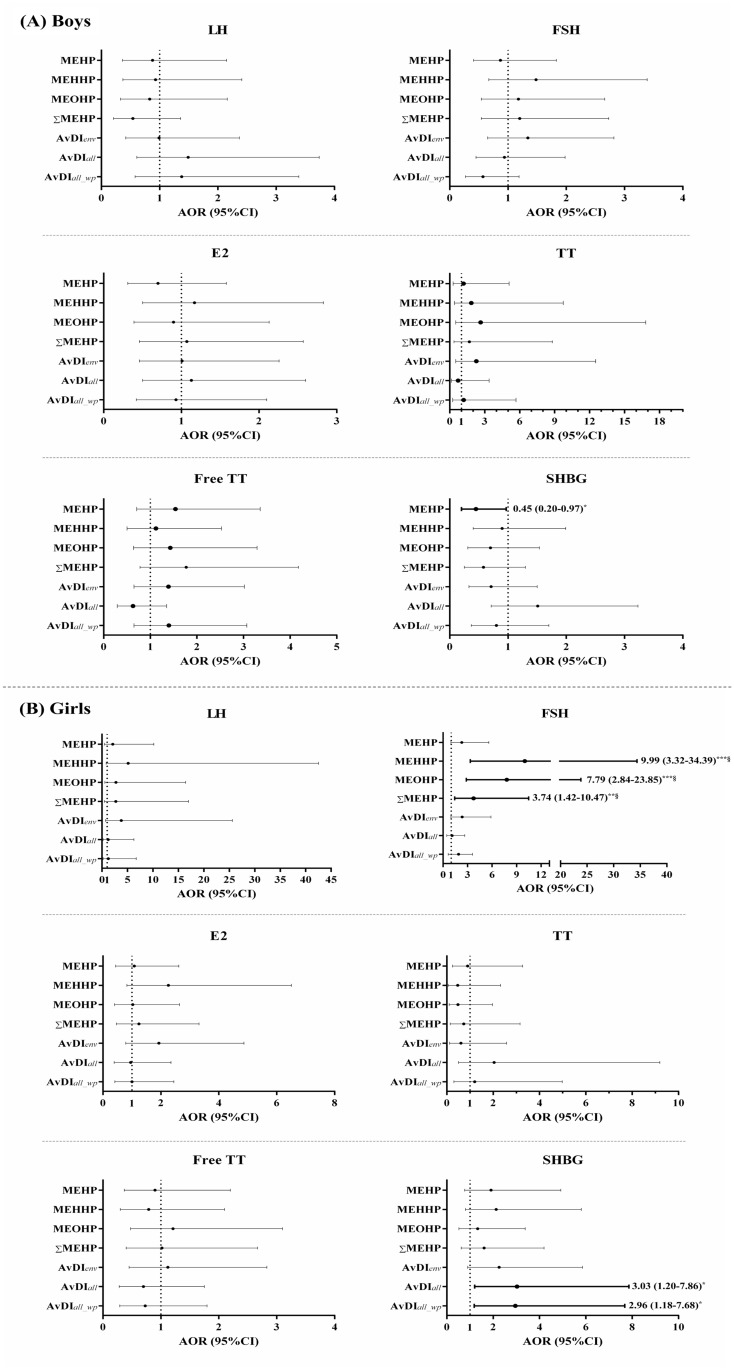
Odds ratios (95% confidence intervals) for the high–low reproductive hormone concentrations between DEHP exposure parameters grouped by median as high and low exposures, with adjustments for age and birth weight (n = 222). The results for (A) boys (n = 132) and (B) girls (n = 90). Numbers in the figures indicated adjusted OR (95% CI). Boldface indicates achievement of *p* < 0.05. *p < 0.05, **p < 0.01, and ***p < 0.001 (^§^*p*<0.0071 indicates a statistical significant). Abbreviations: DEHP, di-(2-ethylhexyl) phthalate; MEHP, mono-(2-ethylhexyl) phthalate; MEHHP, mono-(2-ethyl-5-hydroxyhexyl) phthalate; MEOHP, mono-(2-ethyl-5-oxohexyl) phthalate; AvDI_*all*_, estimated daily intake of DEHP exposure; AvDI_*all_wp*_, AvDIall with window period; LH, luteinizing hormone; FSH, follicle-stimulating hormone; E2, estradiol; TT, testosterone; SHBG, sex hormone-binding globulin.

We then grouped FSH, free TT, and SHBG concentrations by median, and divided LH, E2, and TT concentrations into undetected (80.2%, 66.2%, and 87.8% for LH, E2, and TT, respectively) and detected groups. We also grouped DEHP exposure parameters by median to categorize children into high- and low-exposure groups. The crude odds ratios and 95% confidence intervals (OR [95% CI]) for the high and low reproductive hormone concentrations between DEHP exposure parameters grouped by median are shown in S1 Fig. The association between DEHP exposure and reproductive x hormone levels after adjustments for age and birth weight is shown in Fig 2. After adjustment for age and birth weight, urinary MEHP concentrations above the median were marginally negative association with SHBG concentrations in boys (OR [95% CI] = 0.45 [0.20–0.97], *p* = 0.0446) (Fig 2A). Urinary MEHHP, MEOHP, and ΣMEHP concentrations above the median were positively associated with FSH concentrations in girls (OR [95% CI] = 9.99 [3.32–34.39], *p* < 0.0001) for MEHHP, 7.79 [2.84–23.85], *p* < 0.0001) for MEOHP, and 3.74 [1.42–10.47], *p* = 0.0071) for ΣMEHP) (Fig 2B). Moreover, the estimated AvDI concentration above the median with or without the window period also showed a marginally positive association with SHBG levels in girls (OR [95% CI] = 3.03 [1.20–7.86], *p* = 0.0188) for AvDI_*all*_ and 2.96 [1.18–7.68], *p* = 0.0210) for AvDI_*all_wp*_) (Fig 2B). The association between urinary DEHP metabolites and reproductive hormones was not affected by correction for urinary creatinine concentration, as demonstrated by multiple variable regression analysis using urinary creatinine as an independent variable (S3 Table).

## Discussion

We found that DEHP exposure was associated with altered reproductive hormone levels in girls who were potentially exposed to phthalate-tainted foodstuffs. Higher FSH and SHBG levels were shown in girls with higher current and past DEHP exposure, respectively.

The estimated background DEHP daily intake level (AvDI_*env*_) in our recruited children was reported slightly higher than those in some other countries but lower than those in previous Taiwan studies (Table 4). From a birth cohort study conducted in central Taiwan, the results showed that the geometric mean (GM) of estimating DEHP daily intake was 8.1 and 10.9 μg/kg bw/day in 2-year-old and 5-year-old children, respectively [26]. In Kaohsiung, Wu et al., measured higher DEHP daily intake levels (median: 11.8 μg/kg bw/day) in children aged 1–10 years and recruited immediately as the phthalate episode occurred [27]. In Australia, daily DEHP intake was reportedly higher in children at the age of 6–15 years old than those in our study [28]. However, lower DEHP exposure levels were reported in Greek children aged 2 years old [29] and in German children 5–6 years old [30]. Lower DEHP exposure levels were also reported in the study by Wittassek et al.’s of German children aged 2–14 years [31]. Our children also had higher DEHP exposure levels than did Danish children [32, 33]. Moreover, the percentage of children whose estimated DEHP daily intake dose exceed European Food Safety Authority (EFSA) guideline (50 μg/kg bw/day) in present study was 26.0% for AvDI_*all*_ and 9.4% for AVDI_*all_wp*_, respectively. However, 6 months after the episode, Wu et al. found that the urinary concentration of four phthalate monoesters in children recruited immediately when the phthalate incident occurred was decreased to normal or background levels [27]. Even so, whether children with higher DEHP exposure during the incident have longer-term effects and related health issues still needs to be further investigated.

**Table 4:** Median or geometric mean (GM) of estimated DEHP daily intake levels (μg/kg bw/day) in different countries.

Country	City	Study year	Subjects	Median	Reference
Taiwan	This study	2012–3	≤ 12 years old (n = 222)	^¶^AvDI_*env*_: 6.6 (median) and 6.5 (GM)	
	Taichung	2003–4	2–3 years (n = 30)	8.1	[26]
		2006–7	5–6 years (n = 59)	10.9	
	Kaohsiung	2011	1–10 years (n = 29)	11.8	[27]
Australia	—^&^	2012	6–15 years (n = 387)	7.8	[28]
Greece	Crete	2009–11	2 years (n = 239)	4.0 (median) and 3.3 (GM)	[29]
Germany	Frankfurt	2007	5–6 years (n = 111)	4.5	[30]
	Berlin	2001–2	2–14 years (n = 239)	4.3	[31]
Denmark	—^&^	2006–8	6–10 years (n = 25 for boys, 24 for girls)	Boys: 5.7 Girls: 5.4	[32]
	—^&^	2008–9	3–6 years (n = 431)	4.3	[33]

^¶^AvDIenv: a background daily DEHP exposure for comparison to the other studies.

^&^“—”indicated no information.

In the present study, we found that current DEHP exposures above the median were positively associated with above-median FSH levels in girls who were affected by the phthalate episode. This result was consistent with previous human and animal studies. In a longitudinal follow-up study of 8-year-old children, MEHP correlated significantly with serum FSH levels in girls [10]. Meltzer et al. observed an increase in FSH serum levels in female adult rats following in utero exposure to DEHP [34]. The mechanism underlying the positive association between DEHP exposure and serum FSH levels is still unclear. GnRH that modulates FSH stimulation of the HPG axis was measured at higher levels in rats exposed to higher DEHP concentrations [35, 36]. Moreover, DEHP exposure may regulate the transcription of steroid hormone receptor genes. Ye et al. found that the FSH β subunit gene was upregulated in females exposed to 0.1 mg/L DEHP in marine medaka study [36]. DEHP was also reported to inhibit FSH-stimulated progesterone production [37], and the positive association between DEHP exposure and FSH levels found in the present study may be the positive feedback for the pituitary release of gonadotropic hormones. The unbalanced secretion of FSH may disturb the HPG axis and affect downstream sex hormone secretion and function.

SHBG was also positively associated with DEHP exposure measured by both urinary and estimated levels in girls in the present study. SHBG is a serum glycoprotein mainly produced in the liver, and it binds testosterone and estrogen with high affinity and specificity. SHBG is related to androgen activity because of their ability for binding to TT and therefore regulating the levels of bioavailable free TT. SHBG can be regulated by sex hormones as androgen decreases and estrogen increases SHBG concentration [38, 39]. The positive association of SHBG with DEHP exposure may be due to the anti-androgenic effects of DEHP because testosterone has an inhibitory influence on SHBG [9]. Our results were consistent with anti-androgenic effects of phthalate exposure, and a negative association was found between DEHP and TT concentrations in general, even though the association became statistically non-significant after multiple adjustments due to the limited sample size. Moreover, estrogen is a known SHBG stimulator [39], so elevated SHBG concentrations may also be partially influenced by the estrogenic effects of phthalate exposure.

The strength of this study included recruiting unique participants who possibly were exposed to higher levels of DEHP during the incident in Taiwan. We were able to investigate the effect of high-does DEHP exposure on children’s health in general population. Second, we applied Bayesian models to reconstruct exposure status before the incident occurred using a well-designed exposure questionnaire and urinary measurements.

There are limitations in this study. The cross-sectional study design makes it hard to interpret any causal relationships between DEHP exposure and reproductive hormone levels. However, the results were consistent with previous studies that showed that DEHP had antiandrogenic and estrogenic effect in children [15, 40]. These children should be followed to investigate the effects of exposure to phthalate-tainted foodstuffs on children’s health [41]. Second, the range of the children’s age was wide (mean = 5.48 years; range = 1 to approximately 11.6 years) in the present study. The concentrations of reproductive hormone vary throughout life before puberty [42, 43]. A more narrowly defined age group and a larger sample size should be studied in further investigations. Third, because of the small sample size, we could not consider more potential covariates, such as socioeconomic status, diet, and stress. However, the educational levels of the parents of participating children were almost all at the college level. Factors that closely correlate with parental education levels, such as socioeconomic status and lifestyle [44] may be similar in our cohort of children. Thus, potential confounding by these factors may not be severe in the present study. Fourth, only one measurement of urine and blood samples was done in our study and may not represent the long-term pattern of phthalate exposure and reproductive hormone concentrations. Thus, we assumed that the usage of phthalate-related products was fairly constant in the children’s daily life and that the urinary concentration of phthalate monoesters was therefore representative of their steady-state concentrations. Fifth, we did not investigate the presence of other known environmental endocrine disruptors such as bisphenol A, alkylphenols, or other phthalate esters that may be associated with reproductive hormone concentrations [45, 46]. The effects of multiple environmental exposures on reproductive hormone levels should be investigated in further studies. Sixth, the study was begun one year after the phthalate incident. The participants’ recall bias about using phthalate-tainted products is a concern. Finally, we only had children who possibly were exposed to higher levels of DEHP but without an appropriate control group, and therefore we did not fully determine if such effects were similar (or more adverse) in the general population.

In conclusion, one year after the episode, the exposure to DEHP resulted in altered concentrations of reproductive hormones. In particularly, past DEHP exposure was associated with SHBG in girls. These children should be followed to investigate the long-term effect of exposure to phthalate-tainted foodstuffs on pubertal development.

## Supporting information

S1 Table Spearman's correlation between urinary DEHP monoester and AvDI levels and reproductive hormone concentrations in children (n = 222). (PDF)

S2 Table Spearman's correlation between estimated DEHP exposure levels and reproductive hormone concentrations in children by sex (n = 218). (PDF)

S3 Table The relationship between urinary DEHP monoester and reproductive hormone concentration in children (n = 222). (PDF)

S1 Fig Crude odds ratios (95% confidence interval) for the high-low reproductive hormone concentrations between DEHP exposure parameters grouped by median as high and low exposure (n = 222). The results of (A) Boys (n = 132) and (B) Girls (n = 90).
Bold line indicated achievement of *p* < 0.05.
*p < 0.05, **p < 0.01, and ***p < 0.001 (^§^*p*<0.0071 indicates a statistical significant).
Abbreviations: DEHP, di-(2-ethylhexyl) phthalate; MEHP, mono-(2-ethylhexyl) phthalate; MEHHP, mono-(2-ethyl-5-hydroxyhexyl) phthalate; MEOHP, mono-(2-ethyl-5-oxohexyl) phthalate; AvDI_*all*_, estimated daily intake of DEHP exposure; AvDI_*all_wp*_, AvDIall with window period; LH, luteinizing hormone; FSH, follicle-stimulating hormone; E2, estradiol; TT, testosterone; SHBG, sex hormone-binding globulin.
(TIF)
