# Supplementary material for: Phthalate exposure and reproductive hormones and sex-hormone binding globulin before puberty – Phthalate contaminated-foodstuff episode in Taiwan
Source: PLoS One. 2017 Apr 14;12(4):e0175536. doi: 10.1371/journal.pone.0175536 (PMC5391940; doi:10.1371/journal.pone.0175536)
Supplement: S1 Table — (PDF) [file pone.0175536.s001.pdf]

S1 Table. Spearmen correlation between covariables, DEHP exposure, and reproductive hormones

	All (n = 222)				Boys (n = 132)				Girls (n =90)			
	Age	Gestational week	BMI	Birth weight	Age	Gestational week	BMI	Birth weight	Age	Gestational week	BMI	Birth weight
Urinary DEHP monoesters (μg/g creatinine)												
MEHP	0.025	-0.053	0.0001	-0.07	0.046	-0.07	-0.0032	-0.125	0.053	-0.012	-0.001	-0.01
MEHHP	-0.306***	-0.003	-0.034	0.033	-0.220*	-0.04	-0.019	0.06	-0.409***	0.099	-0.084	-0.029
MEOHP	-0.293***	0.016	-0.05	0.024	-0.222*	-0.01	0.004	0.045	-0.345**	0.096	-0.15	-0.028
∑MEHP	-0.285***	0.004	-0.03	0.018	-0.215*	-0.012	-0.0001	0.033	-0.335**	0.106	-0.119	-0.023
Estimated DEHP exposure levels (μg/kg bw/day)												
AvDI$_{all}$	-0.338***	0.013	-0.321***	-0.142*	-0.348***	0.05	-0.305***	-0.146#	-0.315**	-0.039	-0.351***	-0.137
AvDI$_{all_wp}$	-0.231***	0.012	-0.304***	-0.137*	-0.192*	0.055	-0.249**	-0.162#	-0.309**	-0.05	-0.377***	-0.113
Reproductive hormones												
LH (mIU/mL)	0.289***	-0.041	0.0644	0.056	0.208*	-0.028	0.034	0.013	0.473***	-0.065	0.107	0.138
FSH(mIU/mL)	0.197**	-0.001	0.083	-0.006	0.212*	-0.101	0.14	-0.008	0.061	0.13	0.1	0.137
E2 (pg/mL)	0.124#	0.099	0.059	0.01	0.086	0.083	0.020	-0.003	0.149	0.114	0.094	0.053
TT (ng/dL)	0.360***	0.074	0.201**	0.09	0.330***	0.074	0.182*	0.023	0.392***	0.069	0.234*	0.189#
Free TT (ng/dL)	0.434***	0.116#	0.317***	0.054	0.439***	0.208*	0.247**	0.059	0.403***	-0.043	0.471***	0.091
SHBG (nmol/L)	-0.448***	-0.075	-0.348***	-0.002	-0.468***	-0.187*	-0.293**	-0.023	-0.407***	0.13	-0.458***	0.021

#$p < 0.1$, *$p < 0.05$, **$p < 0.01$, ***$p < 0.001$.
