# Supplementary material for: Phthalate exposure and reproductive hormones and sex-hormone binding globulin before puberty – Phthalate contaminated-foodstuff episode in Taiwan
Source: PLoS One. 2017 Apr 14;12(4):e0175536. doi: 10.1371/journal.pone.0175536 (PMC5391940; doi:10.1371/journal.pone.0175536)
Supplement: S2 Table — (PDF) [file pone.0175536.s002.pdf]

S2 Table. Spearman's correlation between estimated DEHP exposure levels and reproductive hormone concentrations in children by sex (n = 218) ¶

	LH (mIU/mL)		FSH (mIU/mL)		E2 (pg/mL)		TT (ng/dL)		FreeTT (ng/dL)		SHBG (nmol/L)	
	Unadj	adj	Unadj	adj	Unadj	adj	Unadj	adj	Unadj	adj	Unadj	adj
Boys (n = 129)												
$AvDI_{all}$	-0.068	-0.037	-0.129	-0.062	-0.052	-0.031	-0.159[#]	0.001	-0.210*	-0.081	0.219*	0.071
$AvDI_{all\_wp}$	-0.050	-0.055	-0.039	0.068	-0.059	-0.021	-0.111	-0.016	-0.080	-0.043	0.086	0.023
Girls (n = 89)												
$AvDI_{all}$	-0.157	0.035	-0.035	0.045	-0.058	0.001	-0.172	-0.052	-0.266*	-0.094	0.358***§	0.267*
$AvDI_{all\_wp}$	-0.136	0.011	0.011	0.055	-0.070	-0.005	-0.172	-0.043	-0.304**§	-0.122	0.409***§	0.320**§

¶Adjusted for age and birth weight.

#p<0.1, *p<0.05, **p<0.01, ***p<0.001 (§p<0.0071 indicates a statistical significant correlation)

All concentrations of urinary DEHP monoester, estimated DEHP exposure, and reproductive hormones were log 10 transformed.

Some numbers do not add up to total n because of missing values.
