# Supplementary material for: Phthalate exposure and reproductive hormones and sex-hormone binding globulin before puberty – Phthalate contaminated-foodstuff episode in Taiwan
Source: PLoS One. 2017 Apr 14;12(4):e0175536. doi: 10.1371/journal.pone.0175536 (PMC5391940; doi:10.1371/journal.pone.0175536)
Supplement: S3 Table — (PDF) [file pone.0175536.s003.pdf]

S3 Table. The relationship between urinary DEHP monoester and reproductive hormone concentration in children (n = 222)

Urinary DEHP monoesters (µg/L)	LH (mIU/mL)		FSH (mIU/mL)		E2 (pg/mL)	
	n (%) of > LOD	AOR[¶]	n (%) of > median	AOR[¶]	n (%) of > LOD	AOR[¶]
Boys (n = 132)						
MEHP						
<4.87 (n = 63)	17 (27.0)	Reference	25 (39.7)	Reference	23 (36.5)	Reference
≥4.87 (n = 69)	14 (20.3)	0.50 (0.17–1.45)	39 (56.5)	0.91 (0.36–2.25)	12 (17.4)	0.45 (0.16–1.19)
MEHHP						
<20.97 (n = 62)	16 (25.8)	Reference	25 (40.3)	Reference	20 (32.3)	Reference
≥20.97 (n = 70)	15 (21.4)	0.70 (0.22–2.21)	39 (55.7)	0.74 (0.28–1.95)	15 (21.4)	0.97 (0.34–2.80)
MEOHP						
<15.19 (n = 63)	16 (25.4)	Reference	24 (38.1)	Reference	22 (34.9)	Reference
≥15.19 (n = 69)	15 (21.7)	0.75 (0.25–2.29)	40 (58.0)	1.10 (0.43–2.81)	13 (18.8)	0.58 (0.21–1.62)
∑MEHP						
<41.95 (n = 61)	16 (26.2)	Reference	22 (36.1)	Reference	22 (36.1)	Reference
≥41.95 (n = 71)	15 (21.1)	0.66 (0.21–2.09)	42 (59.2)	1.41 (0.53–3.72)	13 (18.3)	0.52 (0.18–1.46)
Girls (n = 90)						
MEHP						
<4.87 (n = 48)	5 (10.4)	Reference	18 (37.5)	Reference	20 (41.7)	Reference

≥4.87 (n = 42)	8 (19.1)	3.10 (0.40–30.59)	25 (59.5)	3.81 (1.36–11.63)*	20 (47.6)	1.64 (0.58–4.74)
MEHHP						
<20.97 (n = 49)	4 (8.2)	Reference	17 (34.7)	Reference	19 (38.8)	Reference
≥20.97(n = 41)	9 (22.0)	4.90 (0.53–59.68)	26 (63.4)	8.51 (2.21–39.01)**	21 (51.2)	2.39 (0.68–9.19)
MEOHP						
<15.19 (n = 48)	4 (4.4)	Reference	15 (31.3)	Reference	19 (39.6)	Reference
≥15.19 (n = 42)	9 (21.4)	4.05 (0.46–43.12)	28 (66.7)	20.10 (4.84–109.05)***	21 (50.0)	2.28 (0.69–8.05)
∑MEHP						
<41.95 (n = 50)	4 (8.0)	Reference	17 (34.0)	Reference	19 (38.0)	Reference
≥41.95 (n = 40)	9 (22.5)	5.10 (0.60–55.45)	26 (65.0)	10.54 (2.85–47.48)***	21 (52.5)	3.28 (0.98–12.17)#

All concentrations of urinary DEHP monoesters and reproductive hormones were log 10 transformed.

#p < 0.1, *p < 0.05, **p < 0.01, ***p < 0.001.

¶Adjusted for age, birth weight, and urinary creatinine concentration.

Some numbers do not add up to total n because of missing values.

Abbreviations: DEHP, di-(2-ethylhexyl) phthalate; MEHP, mono-(2-ethylhexyl) phthalate; MEHHP, mono-(2-ethyl-5-hydroxyhexyl) phthalate; MEOHP, mono-(2-ethyl-5-oxohexyl) phthalate; LH, luteinizing hormone; FSH, follicle-stimulating hormone; E2, estradiol; AOR, adjusted odds ratio; LOD, limit of detection value.

S3 Table (continued). The relationship between urinary DEHP monoester and reproductive hormone concentration in children (n = 222).

Urinary DEHP monoesters (μg/L)	TT (ng/dL)		Free TT (ng/dL)		SHBG (nmol/L)	
	n (%) of > LOD	AOR[¶]	n (%) of > median	AOR[¶]	n (%) of > median	AOR[¶]
Boys (n = 132)						
MEHP						
<4.87 (n = 63)	5 (7.9)	Reference	26 (41.3)	Reference	36 (57.1)	Reference
≥4.87 (n = 69)	7 (10.1)	1.94 (0.32–14.17)	36 (52.2)	1.17 (0.47–2.89)	30 (43.5)	1.08 (0.44–2.72)
MEHHP						
<20.97 (n = 62)	7 (11.3)	Reference	25 (40.3)	Reference	37 (59.7)	Reference
≥20.97 (n = 70)	5 (7.1)	0.60 (0.08–4.40)	37 (52.9)	1.46 (0.56–3.90)	29 (41.4)	0.77 (0.29–2.01)
MEOHP						
<15.19 (n = 63)	6 (9.5)	Reference	26 (41.3)	Reference	37 (58.7)	Reference
≥15.19 (n = 69)	6 (8.7)	1.49 (0.23–11.31)	36 (52.2)	1.21 (0.47–3.12)	29 (42.0)	0.90 (0.35–2.32)
∑MEHP						
<41.95 (n = 61)	6 (9.8)	Reference	26 (42.6)	Reference	35 (57.4)	Reference
≥41.95 (n = 71)	6 (8.5)	1.53 (0.21–12.25)	36 (50.7)	1.02 (0.39–2.71)	31 (43.7)	1.11 (0.42–2.96)
Girls (n = 90)						
MEHP						
<4.87 (n = 48)	9 (18.8)	Reference	21 (43.8)	Reference	25 (52.1)	Reference

≥4.87 (n = 42)	6 (14.3)	0.93 (0.16–5.27)	23 (54.8)	1.00 (0.35–2.79)	20 (47.6)	1.31 (0.47–3.80)
MEHHP						
<20.97 (n = 49)	7 (14.3)	Reference	20 (40.8)	Reference	25 (51.0)	Reference
≥20.97 (n = 41)	8 (19.5)	3.76 (0.54–32.74)	24 (58.5)	1.37 (0.39–4.88)	20 (48.8)	2.17 (0.61–8.27)
MEOHP						
<15.19 (n = 48)	8 (16.7)	Reference	20 (41.7)	Reference	24 (50.0)	Reference
≥15.19 (n = 42)	7 (16.7)	1.19 (0.18–7.58)	24 (57.1)	1.04 (0.31–3.40)	21 (50.0)	2.78 (0.83–10.18)[#]
∑MEHP						
<41.95 (n = 50)	8 (16.0)	Reference	22 (44.0)	Reference	24 (48.0)	Reference
≥41.95 (n = 40)	7 (17.5)	1.44 (0.23–9.16)	22 (55.0)	0.74 (0.22–2.44)	21 (52.5)	3.85 (1.11–15.03)[*]

All concentrations of urinary DEHP monoesters and reproductive hormones were log 10 transformed.

#p < 0.1, *p < 0.05, **p < 0.01, ***p < 0.001.

¶Adjusted for age, birth weight, and urinary creatinine concentration.

Some numbers do not add up to total n because of missing values.

Abbreviation: DEHP, di-(2-ethylhexyl) phthalate; MEHP, mono-(2-ethylhexyl) phthalate; MEHHP, mono-(2-ethyl-5-hydroxyhexyl) phthalate; MEOHP, mono-(2-ethyl-5-oxohexyl) phthalate; TT, testosterone; SHBG, sex hormone–binding globulin; AOR, adjusted odds ratio; LOD, limit of detection value.
