# Supplementary figures and images for: Phthalate exposure and reproductive hormones and sex-hormone binding globulin before puberty – Phthalate contaminated-foodstuff episode in Taiwan
Source: PLoS One. 2017 Apr 14;12(4):e0175536. doi: 10.1371/journal.pone.0175536 (PMC5391940; doi:10.1371/journal.pone.0175536)

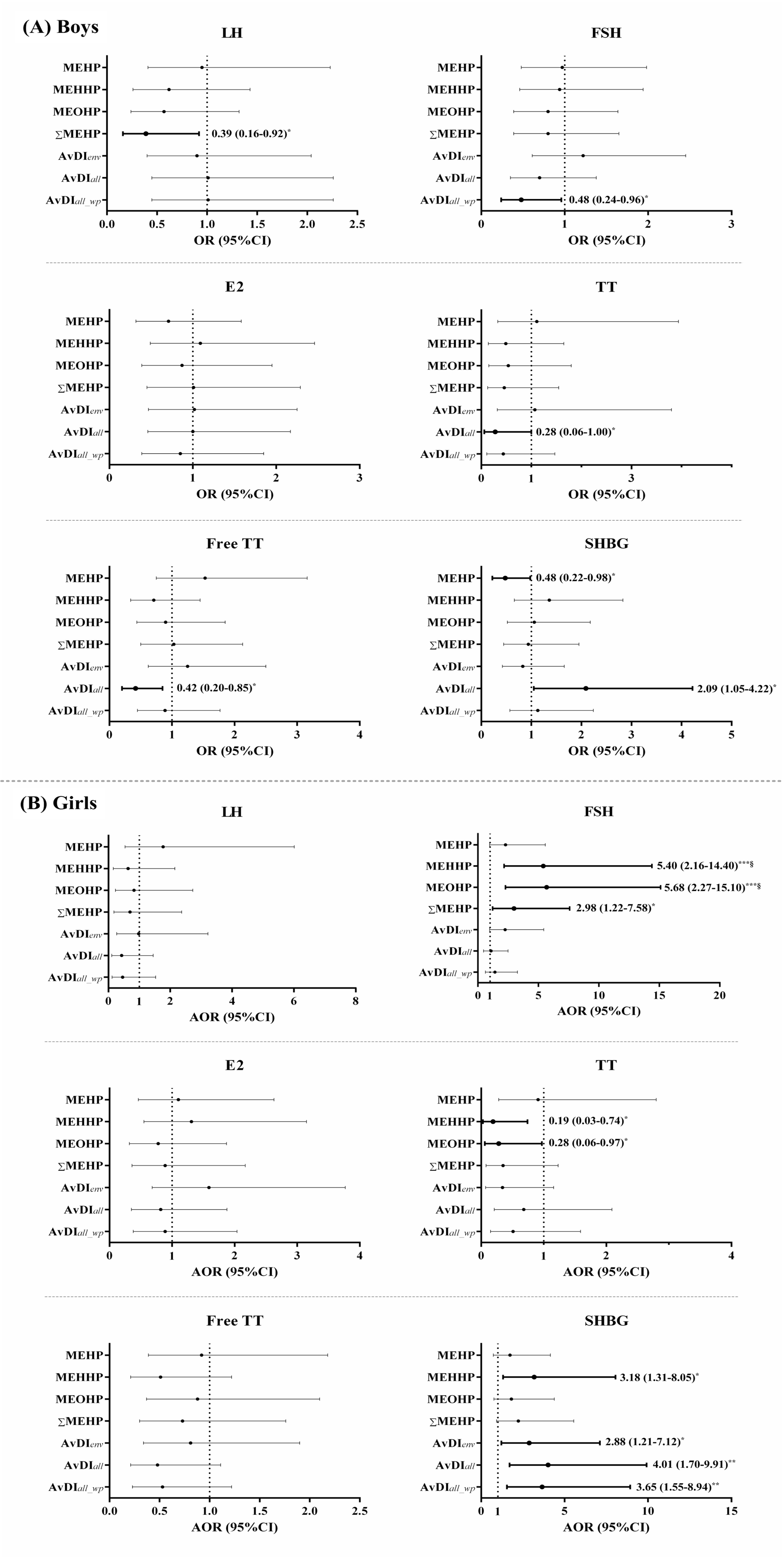

Supplement: S1 Fig — The results of (A) Boys (n = 132) and (B) Girls (n = 90). Bold line indicated achievement of p < 0.05. *p < 0.05, **p < 0.01, and ***p < 0.001 (§p<0.0071 indicates a statistical significant). Abbreviations: DEHP, di-(2-ethylhexyl) phthalate; MEHP, mono-(2-ethylhexyl) phthalate; MEHHP, mono-(2-ethyl-5-hydroxyhexyl) phthalate; MEOHP, mono-(2-ethyl-5-oxohexyl) phthalate; AvDIall, estimated daily intake of DEHP exposure; AvDIall_wp, AvDIall with window period; LH, luteinizing hormone; FSH, follicle-stimulating hormone; E2, estradiol; TT, testosterone; SHBG, sex hormone-binding globulin. (TIF) [file pone.0175536.s004.tif]
